# Differential and directional estrogenic signaling pathways induced by enterolignans and their precursors

**DOI:** 10.1371/journal.pone.0171390

**Published:** 2017-02-02

**Authors:** Yun Zhu, Kayoko Kawaguchi, Ryoiti Kiyama

**Affiliations:** 1 Advanced Biomeasurements Research Group, Biomedical Research Institute, National Institute of Advanced Industrial Science and Technology (AIST), 1-1-1 Higashi, Tsukuba, Ibaraki, Japan; 2 Scinet Company, 4-21-12 Takanawa, Minato-ku, Tokyo, Japan; University of South Alabama Mitchell Cancer Institute, UNITED STATES

## Abstract

Mammalian lignans or enterolignans are metabolites of plant lignans, an important category of phytochemicals. Although they are known to be associated with estrogenic activity, cell signaling pathways leading to specific cell functions, and especially the differences among lignans, have not been explored. We examined the estrogenic activity of enterolignans and their precursor plant lignans and cell signaling pathways for some cell functions, cell cycle and chemokine secretion. We used DNA microarray-based gene expression profiling in human breast cancer MCF-7 cells to examine the similarities, as well as the differences, among enterolignans, enterolactone and enterodiol, and their precursors, matairesinol, pinoresinol and sesamin. The profiles showed moderate to high levels of correlation (*R* values: 0.44 to 0.81) with that of estrogen (17β-estradiol or E_2_). Significant correlations were observed among lignans (*R* values: 0.77 to 0.97), and the correlations were higher for cell functions related to enzymes, signaling, proliferation and transport. All the enterolignans/precursors examined showed activation of the Erk1/2 and PI3K/Akt pathways, indicating the involvement of rapid signaling through the non-genomic estrogen signaling pathway. However, when their effects on specific cell functions, cell cycle progression and chemokine (MCP-1) secretion were examined, positive effects were observed only for enterolactone, suggesting that signals are given in certain directions at a position closer to cell functions. We hypothesized that, while estrogen signaling is initiated by the enterolignans/precursors examined, their signals are differentially and directionally modulated later in the pathways, resulting in the differences at the cell function level.

## Introduction

Mammalian lignans, or enterolignans, are lignans characterized by two phenylpropanoid C_6_-C_3_ units, and two main types of enterolignans, enterolactone (EL) and enterodiol (ED), are found in the urine, plasma, saliva and/or feces of mammals as metabolites of plant lignans, such as matairesinol (MR), secoisolariciresinol, 7ʹ-hydoroxymatairesinol, lariciresinol, isolariciresinol and pinoresinol (PR), and their glycosides [1]. For example, secoisolariciresinol diglycoside and matairesinol glycoside are metabolized by intestinal bacteria to EL, through secoisolariciresinol/ED or MR, respectively [2,3]. Many plant lignans are phytoestrogens, a group of plant chemicals with estrogenic activity. Enterolignans, on the other hand, have been implicated as possessing weakly estrogenic and anti-estrogenic activities and to have various effects on human health, such as protective effects against cancer, osteoporosis and coronary heart disease through their anti-tumor, anti-oxidant and anti-estrogenic properties [1, 4–6]. Reduced risk of breast cancer by modulating estrogen signaling was implicated after the administration of EL and ED, and plant lignans, such as arctiin, sesamin (SE), secoisolariciresinol diglucoside, lariciresinol and tracheloside [7]. Among lignans, SE has been reported as a precursor of enterolignans with protective effects on hormone-related diseases [8].

Estrogen is a sex hormone that plays important roles in various physiological and cellular effects and diseases through estrogen signaling [9]. A comprehensive search of estrogenic chemicals indicates a number of chemicals with structural, functional and original variations, and the pathways involving estrogen signaling vary depending on the types of chemicals, sources/characteristics of cells and conditions of stimulation/signaling [10]. Among the assays for estrogenic activity, gene-expression profiling by means of DNA microarray assay is based on monitoring the estrogenic effects at the transcription level using estrogen-responsive genes with estrogen-receptor (ER) positive cells, and has been applied to a variety of chemicals [11]. A combination of DNA microarray assay with a protein assay, such as Western blotting, for monitoring specific signal mediators enabled us to understand complicated signaling pathways. Especially, the involvement of specific signaling pathways in estrogen signaling would be useful to develop anti-cancer agents and other diagnostic/therapeutic substances [11].

A number of plant lignans modulate estrogen signaling. For example, plant lignans (including their glycosides and derivatives), such as arctigenin/arctiin, guaiacin, *trans*-hinokiresinol, hydroxymatairesinol, 1-hydroxypinoresinol, isoguaiacin, manglieside E, MR, nordihydroguaiaretic acid, nortrachelogenin (wikstromol), nyasol (*cis*-hinokiresinol), oleiferin C, oleiferin D, schizandrin, secoisolariciresinol, silibinin (silybin B) and silymarin (a mixture of flavonolignans), show weak/moderate estrogenic/agonistic activity, while the same or other lignans, such as arctigenin/arctiin, *epi*-aschantin, deoxypodophyllotoxin, eleutheroside E, isolariciresinol, khainaoside, *epi*-magnolin, MR, nectandrin B, nortrachelogenin, PR, princepin, savinin, schizandrin B, SE, silibinin, syringaresinol, tracheloside, *epi*-yangambin and yatein, show weak/moderate anti-estrogenic/antagonistic activity (summarized in Kiyama, 2016 [12]). On the other hand, enterolignans, such as dihydroenterolactone, ED and EL, are known to show estrogenic activity [13–17]. Because of their estrogenic/anti-estrogenic activity, these lignans are often utilized for food, supplements, diagnostics and medicines. However, how to predict the estrogenic/anti-estrogenic effect of lignans is not well understood. To further explore the applications of estrogenic lignans, it is essential to understand the mechanism of action, especially at the cell signaling level [12].

We examined here the estrogenic activity of enterolignans and their precursors along with the signaling pathways involved in this action.

## Materials and Methods

### Antibodies and reagents

Rabbit antibodies against human cyclins D1 (#2978) and E (#4132), and cyclin-dependent kinase 4 (CDK4) (#12790), were obtained from Cell Signaling Technology (Danvers, MA). A mouse monoclonal antibody against human β-actin (#ab6276) was obtained from Abcam (Cambridge, United Kingdom). A horseradish peroxidase (HRP)-linked goat antibody against rabbit IgG (#7074) and a horse antibody against mouse IgG (#7076) were obtained from Cell Signaling Technology. 17β-estradiol (E_2_; #E1132), SE (#S9314), ED (#45198), EL (#45199), MR (#40043), and PR (#40674) were obtained from Sigma-Aldrich (St. Louis, MO). ICI 182,780 was obtained from Tocris Bioscience (Bristol, United Kingdom). LY294002 was obtained from EMD Millipore (Billerica, MA).

### Sulforhodamine B (SRB) assay

Human breast cancer MCF-7 cells were cultured in a phenol red-free RPMI 1640 medium (Life Technologies, Carlsbad, CA) supplemented with 10% fetal bovine serum (FBS) at 37°C in a humidified atmosphere containing 5% CO_2_. The SRB assay was performed to examine cell proliferation as described previously [18]. Briefly, MCF-7 cells were treated with medium containing 10% dextran-coated charcoal-treated FBS (DCC-FBS) for 3 days. After treatment with 10 nM E_2_ or 10 nM to 100 μM of the indicated chemicals for 3 more days, the cells were fixed and stained with 0.4% SRB (Sigma-Aldrich). The bound protein was dissolved with 10 mM Tris-base and transferred into 96-well plates to measure the absorbance at 490 and 650 nm. Three independent assays were performed for each treatment and the data were analyzed by *t*-test.

### DNA microarray assay

Preparation of cells, RNA preparation and cDNA labeling, followed by a focused oligo-DNA microarray assay, were performed as described previously [18]. After treatment with DCC-FBS, MCF-7 cells were incubated with 10 nM E_2_ or 10 μM of each lignan for 3 days. Cells treated with 0.1% DMSO (vehicle) were used as a control. While the oligo-DNA microarray contained a total of 203 genes including 172 estrogen-responsive genes, we used a total of 150 genes selected from the 172 genes based on the reproducibility [19], and calculated the normalized signal intensity for each gene as the ratio of the mean signal intensity for a chemical-treated sample to that for an untreated sample. The ratios of signal intensity for all genes were normalized against the mean ratio for the 28 control genes, and the normalized ratios were log_2_-transformed and used for correlation analysis [20]. A coefficient of correlation between gene expression profiles (*R*-value) was calculated based on linear regression. Functional cluster analysis was performed according to Inoue et al. (2007) [21]. Gene functions are based on the Gene Ontology terms in the Entrez Gene database (www.ncbi.nlm.nih.gov/entrez/). The microarray data are available in the Gene Expression Omnibus database (www.ncbi.nlm.nih.gov/geo/) with Accession No. GSE86565.

### Western blotting

Before stimulation of MCF-7 cells with chemicals, the cells were plated in a phenol red-free RPMI 1640 medium containing 10% DCC-FBS on 6-cm plates at a density of 10^5^ cells per well, cultured for 2 days and then cultured for one more day in a serum-free medium. After the cells had been pretreated with 1 μM ICI 182,780 for 1 h, or 50 μM LY294002 for 30 min, they were treated with 10 nM E_2_, 10 μM of each lignan or vehicle (0.1% v/v DMSO) for the indicated times. Total protein was extracted from the cells and examined by SDS-PAGE using a 5–20% gradient gel, and after the proteins was electro-transferred onto nitrocellulose membranes using a semi-dry transfer cell (Bio-Rad Laboratories, Benicia, CA), they were analyzed with indicated antibodies. The antigen-antibody complex was detected with HRP-coupled goat antibodies against rabbit IgG, and visualized using the Immobilon Western Chemiluminescent HRP Substrate (EMD Millipore).

### MCP-1 immunoassay

MCF-7 cells were treated with a medium containing 10% dextran-coated charcoal-treated FBS (DCC-FBS) for 3 days. After treatment with 10 nM E_2_, 10 μM of the indicated chemicals or chemicals were mixed with 1 μM ICI 182,780 for 3 more days, culture supernatants were recovered and concentrated using Vivaspin (GE Healthcare, Marlborough, MA). The levels of MCP-1 were measured by enzyme immunoassay (R&D Systems, Minneapolis, MN) according to the manufacturer’s instructions. Three independent assays were performed for each treatment and the data analyzed by *t*-test.

## Results

### Lignans and cell proliferation

**Fig 1:**
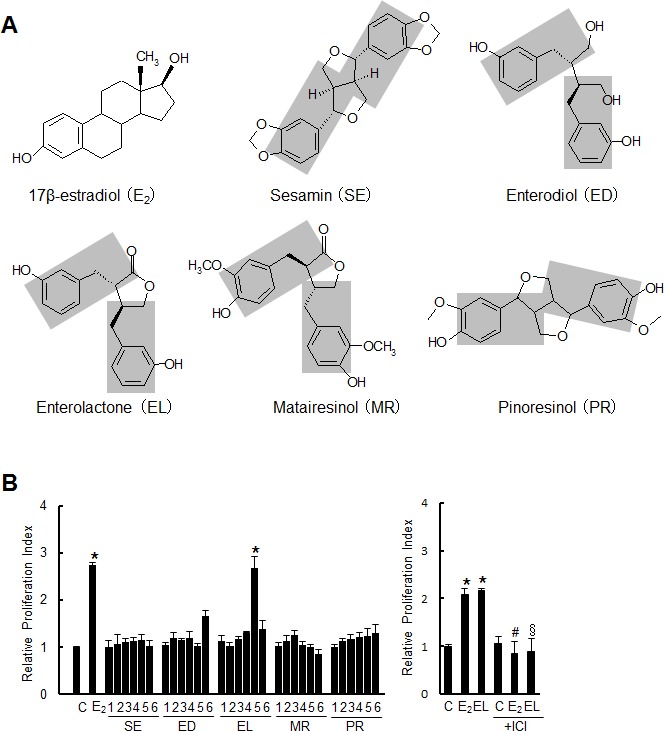
**Chemical structure (A) and cell-proliferation assay (B) for lignans.** (**A**) The phenylpropane backbone is shadowed. (**B**) MCF-7 cells were treated with vehicle (dimethylsulfoxide, DMSO), E_2_ (10 nM) or different concentrations of chemicals as indicated: 1, 1 nM; 2, 10 nM; 3, 100 nM; 4, 1 μM; 5, 10 μM; and 6, 100 μM (on the left), and 10 μM EL (on the right). After incubation for 72 h, cell proliferation was examined by sulforhodamine B (SRB) assay. The rates of cell proliferation in response to E_2_ or lignans to that of a control (DMSO) are shown in the graph. *: *p* < 0.05; vs. control (C), #: *p* < 0.05; vs. E_2_, or §: *p* < 0.05; vs. EL. ICI: ICI 182,780, an ER antagonist.

To understand the estrogenic activity of enterolignans, we first examined the effect of enterolignans, enterodiol (ED) and enterolactone (EL), and their precursors, sesamin (SE), matairesinol (MR) and pinoresinol (PR) (Fig 1A), on the proliferation of ER-positive human breast cancer MCF-7 cells (Fig 1B, left panel). Only EL showed an enhancement of cell proliferation equivalent to that for E_2_ at a statistically significant level (lane 5 for EL, Fig 1B, left panel), while the other enterolignans/precursors did not show such an enhancement. When the effect of anti-estrogen ICI 182,780 was examined, it inhibited the proliferation of MCF-7 cells induced with EL, exactly as observed for E_2_ (Fig 1B, right panel), suggesting that the activation of cell proliferation with EL involves ER signaling.

### Lignan-dependent gene expression profiles

**Fig 2:**
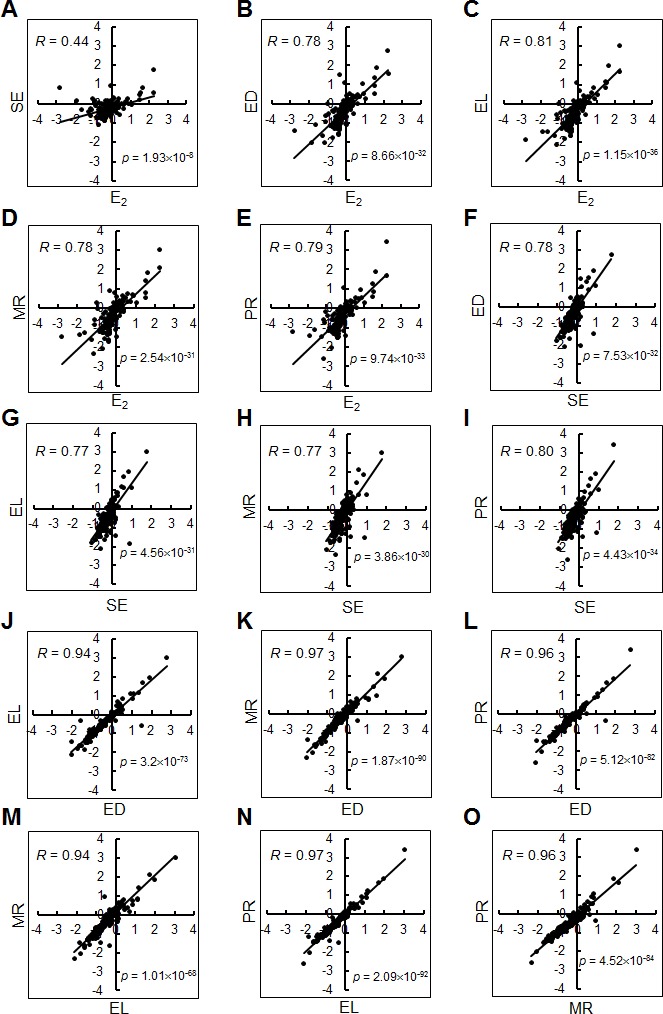
Estrogenic gene expression profiles of lignans revealed by DNA microarray assay. Correlation of gene expression profiles was examined between individual pairs of E_2_ and lignans. The gene expression profiles for these chemicals were compared using a set of 150 estrogen-responsive genes in scatter-plot graphs. The vertical and horizontal axes indicate log_2_ values of the signal intensities. *R*- and *p*-values were calculated for each graph on the basis of linear regression between two profiles.

We then examined the gene expression profiles for the enterolignans/precursors by means of DNA microarray assay using estrogen-responsive genes [11]. The DNA microarray used contained a set of 172 estrogen-responsive genes from a total of more than 20,000 human genes and has been used to examine the estrogenicity of chemicals, such as industrial/natural estrogens, phenolics, micoestrogens, phytoestrogens, environmental pollutants and toxicological estrogens [11]. Estrogenicity is examined by the similarity of the gene expression profiles between the test compounds and a standard estrogen, 17β-estradiol (E_2_), and evaluated by their correlation coefficients (or *R*-values) based on linear regression for the correlation of their profiles. We examined the gene expression profiles after the treatment with 10 μM lignans, under which there was no cytotoxic effect and EL showed differential cell proliferation activity from other lignans. The *R*-values for the profiles between the respective enterolignans/precursors and E_2_ were 0.44 (SE), 0.78 (ED), 0.81 (EL), 0.78 (MR) and 0.79 (PR) (Fig 2A to 2E). We also examined the *R*-values for pairs among the enterolignans/precursors examined (Fig 2F to 2O; S1 Table). Of all the combinations examined, those among ED, EL, MR and PR showed high levels of correlation (*R*-values: 0.94 to 0.97). The combinations of SE or E_2_ with other enterolignans/precursors showed relatively low levels (*R*-values: 0.77 to 0.81), and the combination between SE and E_2_ was lowest (*R*-value: 0.44).

We also examined the gene expression profiles using six functionally designated sets of genes (representing enzymes, signaling, proliferation, transcription, transport and others) (S1 Fig). The genes used were grouped according to their functions designated in the Entrez Gene database (see Kiyama and Zhu, 2014 [11]; Kiyama et al., 2014 [22]) and used for profiling. While most categories showed relatively high levels of correlation (*R*-values: greater than 0.8), the genes related to transcription showed less (*R*-values: 0.4 to 0.6). SE showed very low levels in all categories, and only the category of signaling showed statistically significant correlation.

### Lignans and cell signaling

**Fig 3:**
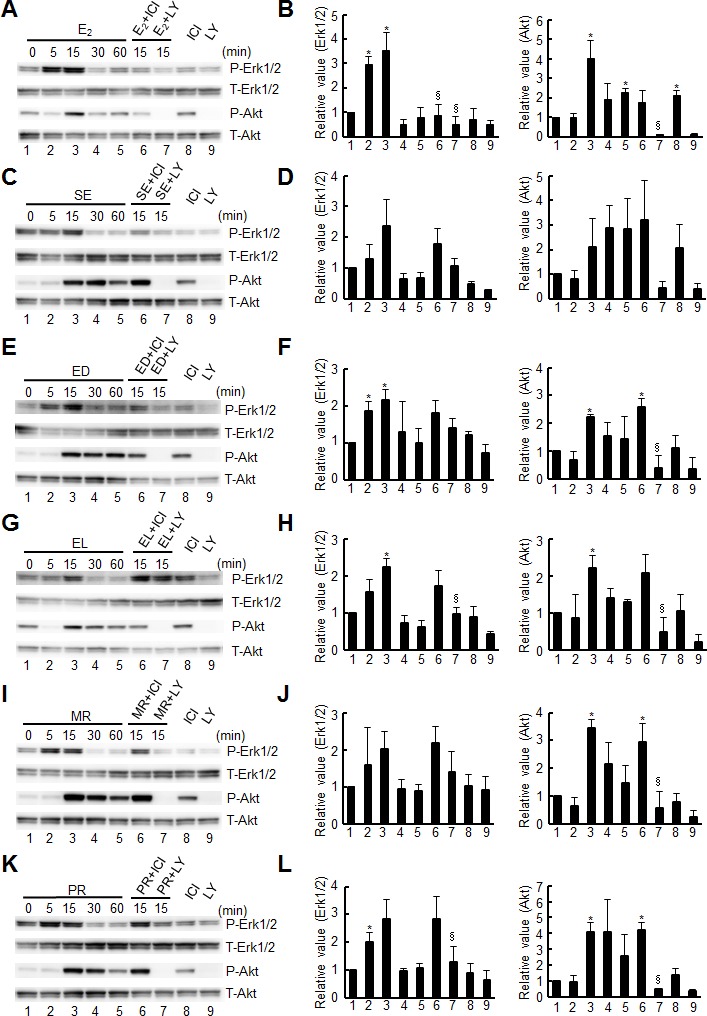
Western-blot analysis of Erk1/2 and Akt signaling pathways induced by lignans. Active/total Erk1/2 and Akt were analyzed by Western blotting. MCF-7 cells were treated with 10 nM E_2_ (**A**) or 10 μM each of lignans (**C**, **E**, **G**, **I**, and **K**) in the presence or absence of inhibitors, ICI 182,780 (ICI) or LY294002 (LY), for the indicated times (minutes), and cell extracts were subjected to Western blot analysis for phosphorylated (P-) or total (T-) proteins as indicated. The results of three independent experiments are summarized along with the statistical evaluation in panels **B** (for E_2_), and **D**, **F**, **H**, **J**, and **L** (for lignans). Statistical significance of data compared with the negative (lane 1) or positive (lanes 3) controls is shown as * (*p* < 0.05; vs. lane 1) or § (*p* < 0.05; vs. lane 3).

We then examined how the estrogen signaling pathways are modulated by the stimulation with enterolignans/precursors. To exclude the genomic pathway of estrogen signaling, we examined a rapid (within 30 min) signaling pathway by monitoring the phosphorylation of signal mediators, Erk1/2 and Akt (Fig 3). Erk1/2 and Akt are known as key signal mediators in the mitogen-activated protein kinase (MAPK) or phosphatidylinositol-3-kinase (PI3K) signal transduction pathways, respectively, and their activation, or phosphorylation, was examined to monitor the non-genomic pathway of estrogen signaling [10]. All enterolignans/precursors examined showed a rapid response of cells after their stimulation (Fig 3C, 3E, 3G, 3I and 3K), which was as expected for E_2_ (Fig 3A). Most of the results showed changes at statistically significant levels (*p* < 0.05) (Fig 3F, 3H and 3L), while some showed less, but good, statistical levels (Fig 3D and 3J). As for the treatment with inhibitors against ERs (ICI 182,780) or PI3K (LY294002), the former did not work (lane 6), while the latter worked (lane 7), suggesting the involvement of the PI3K/Akt pathway but not ERs directly in the rapid signaling. All the enterolignans/precursors examined thus showed rapid responses in the Erk1/2 and PI3K/Akt pathways, although the involvement of ERs is not clear.

### Lignans and cell functions

**Fig 4:**
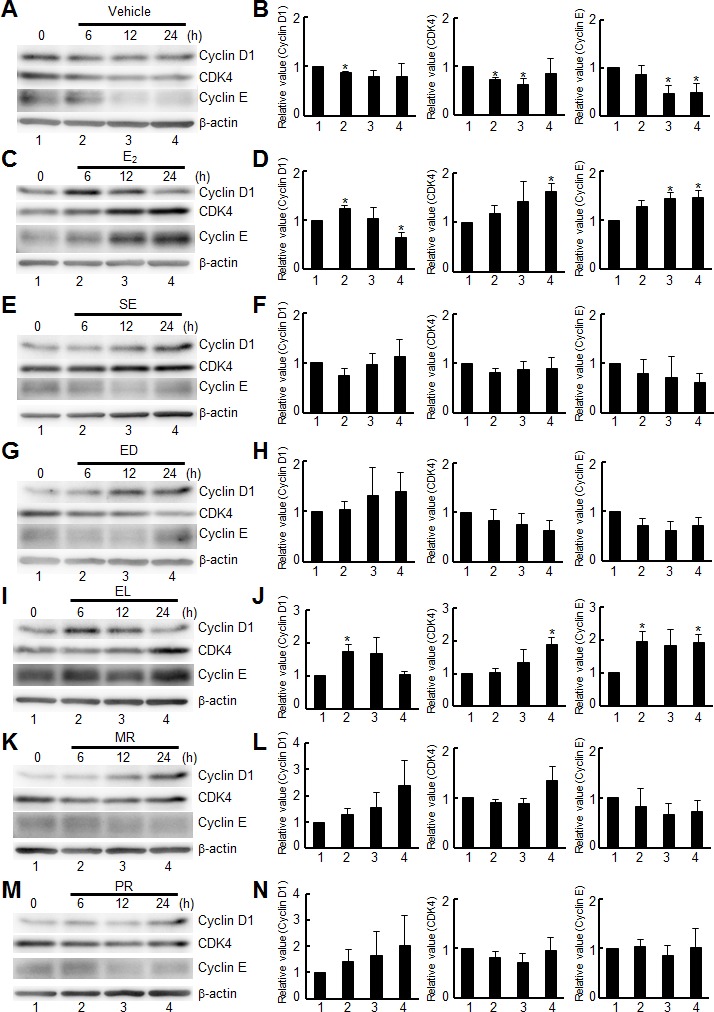
Differential regulation of cell cycle by lignans. MCF-7 cells were treated with vehicle (**A**) or 10 nM E_2_ (**C**), or 10 μM each of lignans (**E**, **G**, **I**, **K**, and **M**) for the indicated times. Cell extracts were subjected to Western blot analysis for cyclin D1, CDK4, cyclin E and β-actin (control). The results of three independent experiments are summarized along with the statistical evaluation in panels **D** (for E_2_), and **F**, **H**, **J**, **L** and **N** (for lignans). Statistical significance of data compared with the negative control (lane 1) is shown as * (*p* < 0.05).

**Fig 5:**
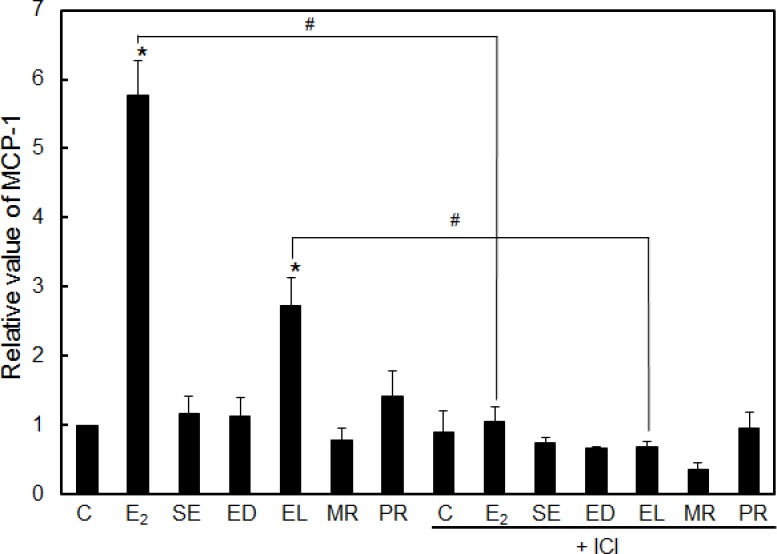
Secretion of MCP-1 in response to stimulation with lignans. MCF-7 cells were treated with E_2_ (10 nM) or each of the lignans (10 μM) for 72 h in the presence or absence of ICI 182,780 (ICI). Then, the supernatants were collected and ELISA was performed according to the manufacturer's instructions. The data represent the mean ± SD of three independent experiments. *: *p* < 0.05; vs. control (C), or #: *p* < 0.05; vs. ICI 182,780 (-).

We then asked whether the cell signaling for enterolignans/precursors is similar to that for estrogen by examining two different cell functions, cell cycle progression and chemokine secretion (Figs 4 and 5). First, to understand the effect of lignans on cell cycle, we examined several cell cycle regulators, cyclins D1 and E and CDK4 (Fig 4), which have been known to regulate the cell cycle of breast cancer cells under estrogen stimulation [23]. Cyclin D1 acts as an oncogene in breast cancer by giving selective advantages to cancer cells, such as activation of its partner CDK4 and other cell cycle regulators for deregulating normal pathways and leading to abnormal cell cycle progression [24]. Another pathway involving cyclin E/CDK2 also contributes to cell cycle progression in breast cancer cells [23]. E_2_ activates both cyclin D1/CDK4 and cyclin E/CDK2 complexes during G1-S phase progression, and while the expression of cyclin D1 reaches maximum around 8–12 h after E_2_ stimulation in MCF-7 cells and then shows a decrease, the expressions of CKD4 and cyclin E continuously increase during 2–16 h after E_2_ stimulation without, or with little, declination at 24 h [25, 26]. In our analysis, while there was no change of cyclin D1 in the control experiment (vehicle; Fig 4A and 4B), a statistically significant increase was observed at 6 h after the treatment with 10 nM E_2_ (lane 2, Fig 4C and 4D), suggesting cell cycle progression. Additionally, E_2_-specific increases reported previously were also observed in our analysis of CDK4 and cyclin E (lanes 3 and 4, Fig 4C and 4D). Similarly, only EL showed a statistically significant increase in the levels of cyclin D1at 6 h (lane 2, Fig 4I and 4J), or CDK4 and cyclin E at 24 h after stimulation (lane 4, Fig 4I and 4J) among the lignans examined, suggesting that cells responded only to EL for cell cycle progression.

Second, we examined the effect of enterolignans/precursors on chemokine secretion. It is known that MCF-7 cells secrete a chemokine, MCP-1 (monocyte chemoattractant protein-1), or chemokine (C-C motif) ligand 2 (CCL2), which belongs to the CC chemokine family and functions by recruiting monocytes and leukocytes to the sites of inflammation in atherogenesis [27]. MCP-1 induces tumorigenesis of breast cancer by stimulating epithelial-mesenchymal transition (EMT) and cell migration, which are mediated by the ERK/GSK-3β/Snail pathway [28]. Significant increases in the secretion of MCP-1 were observed after the treatment of cells with E_2_ or EL, but these were cancelled by co-treatment with an ER antagonist, ICI 182,780 (Fig 5). The results shown here suggest that only EL activates cell functions such as those related to cell proliferation (Fig 1B), cell cycle progression (Fig 4), and chemokine secretion (Fig 5), partly through estrogen signaling.

## Discussion

### Modulations of gene-expression and cell signaling with enterolignans and their precursor

Enterolignans, such as EL and ED, have been implicated in various effects, such as a reduced risk of cardiovascular disease/atherosclerosis [29, 30], obesity [31], prostate cancer [32], breast cancer [33–36], and colorectal adenoma [37]. After modifications by intestinal bacteria, they are absorbed, delivered into blood, and transported to cells to perform various functions [38, 39]. Lignans are known to be associated with various cell functions through specific signaling pathways [12]. In the present study, we explored the differences among lignans, especially between enterolignans and their precursors, by examining gene-expression profiles and cell signaling pathways. Gene-expression profiling did not show clear differences among enterolignans and their precursors (*R*-value = 0.94 to 0.97) except SE (Fig 2 and S1 Table). SE showed relatively low *R*-values when it was compared with E_2_ (0.44) or other lignans (0.77 to 0.80), probably because of the absence of the hydroxyl group; only SE lacks the group. The hydroxy groups in lignans contribute to estrogenic activity (see Kiyama, 2016 [12]). Therefore, gene functions may be expected to be the same among enterolignans and their precursors at the transcription level. However, there were differences at the level of cell functions, such as cell proliferation (Fig 1) and cell signaling related to cell cycle progression and chemokine secretion (Figs 4 and 5). Only EL showed activation of cell cycle-related regulators (Fig 4) and MCP-1 secretion (Fig 5). Thus, we hypothesized that the signals induced by enterolignans/precursors are differentially and directionally modulated later in the pathways related to specific functions. Note that estrogenic activity of lignans can be demonstrated by the sensitivity of cells to ER antagonists such as ICI 182,780 [40, 41], although receptor crosstalk [42] involved in the signaling would mask the sensitivity as was shown here.

### Estrogenic potential of enterolignans and their precursors

**Fig 6:**
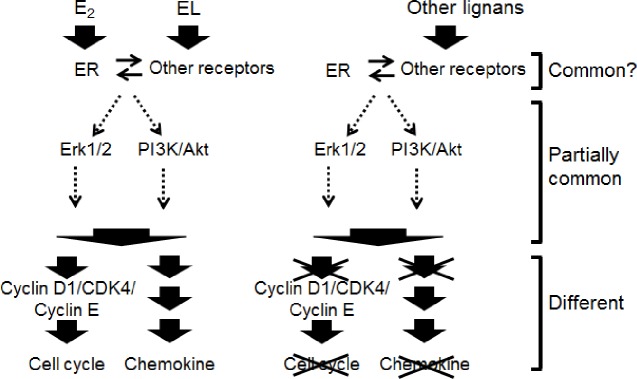
Potential signaling pathways induced by lignans.

Plant lignans are considered important supplements for cardiovascular benefits due to their estrogenic activity, and they may be more important than isoflavones as a source of nutrition [43]. Thus, it is essential to unravel the mechanism of action for the beneficial use of lignans, especially enterolignans, based on their estrogenic activity, because they are closer to the form and the site of action than their precursors. Two major pathways related to estrogen signaling, the Erk1/2 and PI3K/Akt pathways, were examined, as was E_2_. The results showed that they were almost equally stimulated by each of the enterolignans/precursors examined, although they showed differences in cell functions. We summarized the potential pathways of rapid signaling leading to cell functions examined (Fig 6). As discussed above, the signals induced with enterolignans/precursors show similarity at the early stage, but are differentially and directionally modulated later in the pathways related to cell proliferation, cell cycle progression and chemokine secretion. These differences could be explained by the nature of each of the enterolignans/precursors. How these receptors, signal mediators, pathways and outcomes (cell cycle regulation and chemokine secretion for example) are connected will be an important subject of the future signal transduction study by combinations of hypothetical pathways made by the transcriptomic study (see Tanji and Kiyama, 2004 [44]) and the study of signal transducers/mediators at the protein level (as shown here).

Enterolignans have been reported to show estrogenic activity (see Introduction). However, they are also associated with anti-estrogenic activity, or act as a selective estrogen-receptor modulator (SERM), which shows tissue-specific agonistic activity [40, 45]. These contradictory results have been seen for other estrogenic chemicals although the reason is not always clear [12]. Furthermore, while EL activates estrogen-responsive genes, such as *cyclin D1* and *Ki67*, through direct binding to the ligand-binding domains of ERα [40], it also binds to other proteins and may modulate cell functions. For example, the binding of EL and ED to the hydrophobic pocket of human serum albumin may change their activities [46]; EL is a competitive inhibitor of ABCG2, an ATP-binding cassette (ABC) transporter [47]; and, the binding of EL to other proteins, such as sex hormone binding globulin, sex steroid binding protein and α-fetoprotein, was also reported [4], suggesting the presence of additional activities. However, there are differences between ED and EL, such as in the mechanism of ERα transcriptional activation; EL acts through the activation function-2 (AF-2) of ERα, while ED acts through both AF-1 and AF-2 [41]. Most phytoestrogens, including lignans, have estrogenic activity 1/1000 or less, compared with E_2_ [48]. As a result, as the estrogenic activity of each chemical is lower, the relative activity to stimulate other receptors could become greater, revealing additional activities. To understand the complex nature of enterolignans and to explore their applications for medicines and therapeutics, it is essential to understand their mechanism of action at the level of cell signaling.

## Conclusion

We reported previously a comprehensive analysis of estrogenic lignans through a literature search [12]. Here, we used DNA microarray assay along with cell signaling pathway analysis at the protein level to examine similarities and differences among several lignans, including enterolignans, and found that several key genes (proteins) showed differences, while many estrogen-responsive genes showed similarities. Pathway-based functional analysis, such as that shown here, will become more important in understanding the complex nature of lignans and other useful phytochemicals.

## Supporting Information

S1 Fig Evaluation of estrogenicity of lignans based on quantitative profiling of functional groups. Bars indicate the correlation coefficients (*R*-values) between E_2_ and each of the chemicals for the 150 gene set or for the genes categorized into six groups (see Panel **A**). Statistical significance of *R*-values was evaluated using *p*-values, where **: *p* < 0.01.
(TIF)

S1 Table Pair-wise comparison of the gene expression profiles among the lignans and E_2_. (TIF)
