# Supplementary figures and images for: Differential and directional estrogenic signaling pathways induced by enterolignans and their precursors
Source: PLoS One. 2017 Feb 2;12(2):e0171390. doi: 10.1371/journal.pone.0171390 (PMC5289560; doi:10.1371/journal.pone.0171390)

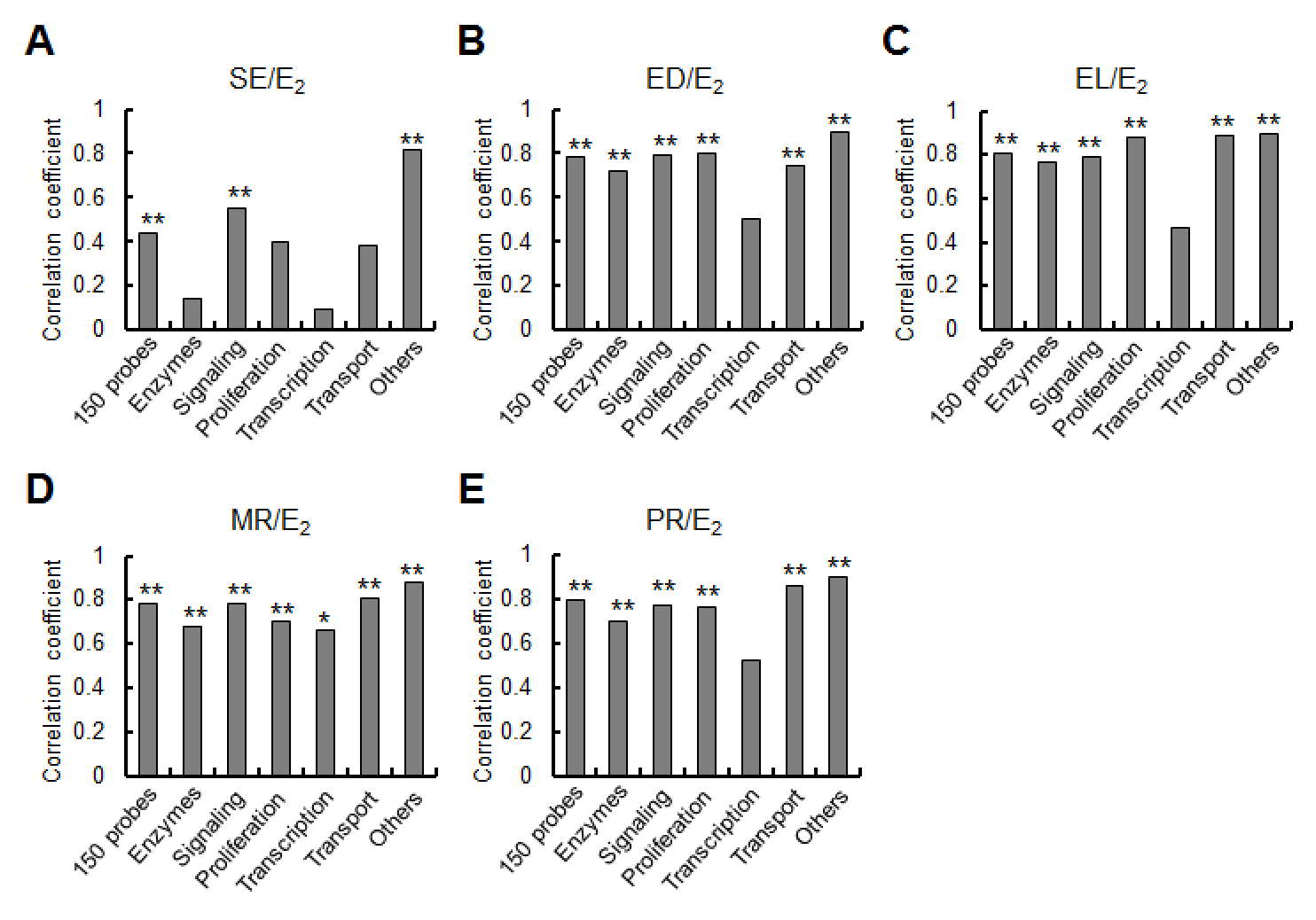

Supplement: S1 Fig — Bars indicate the correlation coefficients (R-values) between E2 and each of the chemicals for the 150 gene set or for the genes categorized into six groups (see Panel A). Statistical significance of R-values was evaluated using p-values, where **: p < 0.01. (TIF) [file pone.0171390.s001.tif]

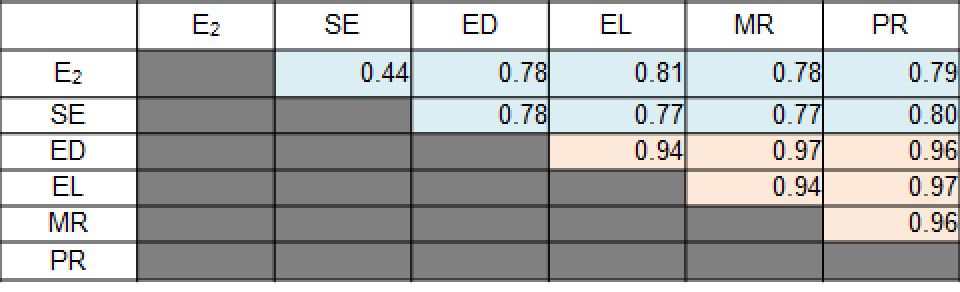

Supplement: S1 Table — (TIF) [file pone.0171390.s002.tif]
